# At the Fulcrum in Health and Disease: Cdk5 and the Balancing Acts of Neuronal Structure and Physiology

**DOI:** 10.4172/2168-975X.S1-001

**Published:** 2012-07

**Authors:** Kristina A. McLinden, Svetlana Trunova, Edward Giniger

**Affiliations:** 1 National Institute of Neurological Disorders and Stroke, USA; 2 National Human Genome Research Institute, USA

**Keywords:** Cdk5, Neuronal structure, Synapse, Dendrite development, Cyclin-dependent kinase

## Abstract

Cdk5 has been implicated in a multitude of processes in neuronal development, cell biology and physiology. These influence many neurological disorders, but the very breadth of Cdk5 effects has made it difficult to synthesize a coherent picture of the part played by this protein in health and disease. In this review, we focus on the roles of Cdk5 in neuronal function, particularly synaptic homeostasis, plasticity, neurotransmission, subcellular organization, and trafficking. We then discuss how disruption of these Cdk5 activities may initiate or exacerbate neural disorders. A recurring theme will be the sensitivity of Cdk5 sequelae to the precise biological context under consideration.

## Introduction

Cyclin-dependent kinase 5 (Cdk5) is a protein serine, threonine kinase that regulates neuronal migration, neurite extension and compartmentalization [1–3], both pre- and post-synaptic aspects of neurotransmission [4,5] and synaptic plasticity [6]. Because of its roles in development and maintenance of neuronal structure and in synaptic plasticity, Cdk5 is essential for higher neural functions such as memory [7]. Dysfunction of Cdk5 is associated with a broad range of neurological disorders including neurodegenerative diseases such as Alzheimer’s Disease (AD) [8], Parkinson’s Disease (PD) [9], Amyotrophic Lateral Sclerosis (ALS) [10] and Huntington’s Disease (HD) [11], and brain disorders such as ischemia and stroke [12], epilepsy [13] and attention deficit disorders [14], among others.

In this review, we summarize the roles that Cdk5 plays in synaptic homeostasis, plasticity, neurotransmission, synaptic position, and intracellular trafficking, with an eye to gaining insight into how disruption of these activities of Cdk5 contributes to neural disease. A common theme throughout this review will be the seemingly contradictory effects of Cdk5 on various aspects of neuronal activity and structure in different experimental systems, reflecting the remarkable dependence of Cdk5 action upon the cell type, age, homeostatic set point and other aspects of the biological context of the neuron under study.

## Properties of Cdk5

**Figure 1:**
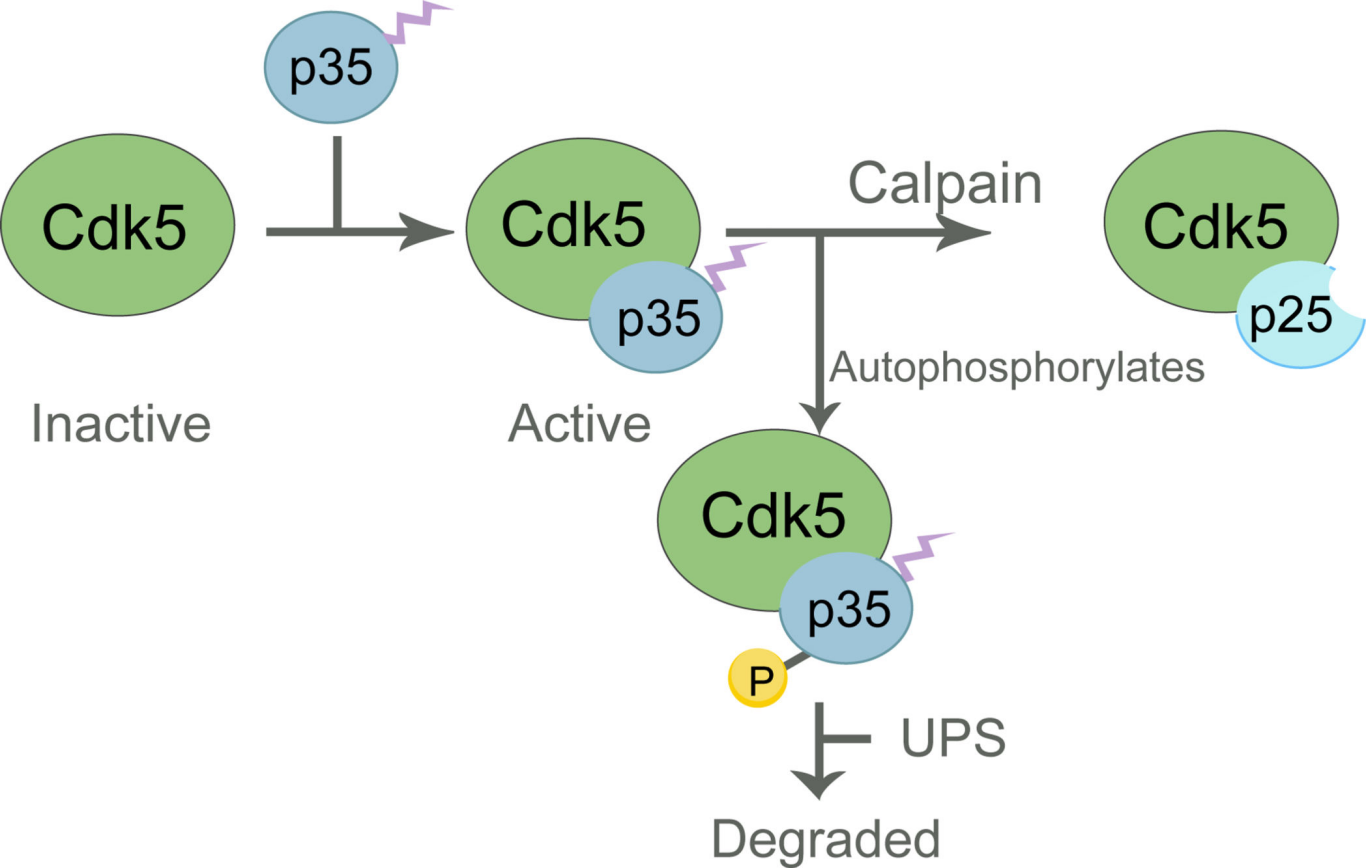
The lifecycle of Cdk5 Cdk5 kinase activity is initiated by binding to the myristoylated regulatory subunit, p35. Myristoylation links Cdk5/p35 to the plasma membrane, limiting the action of the complex to specific cellular compartments. Cdk5/p35 autophosphorylates, leads to ubiquitylation and degradation. Alternatively, p35 can be cleaved by calpain in response to elevated cytosolic Ca^+2^ to produce Cdk5/p25. This form is resistant to degradation, and it lacks the myristoylation site, allowing the complex to dissociate from the membrane and interact with different targets.

Cdk5 is a member of the cyclin-dependent kinase family of protein kinases, closely related to the Cdk regulators of the cell division cycle. Unlike other members of the Cdk family, however, Cdk5 is not activated by canonical cyclins [15], and does not play a significant role in the cell cycle [16]. Instead, Cdk5 activity is largely limited to post mitotic neurons due to the absolute requirement that it bind one of two paralogous neuron-specific regulatory subunits, p35 or p39 [17,18] (Figure 1). While p35 and p39 have little or no sequence homology to traditional cyclins, they assume the three-dimensional “cyclin fold” [19]. Consequently, upon association with Cdk5, they induce a conformational change in the catalytic subunit similar to that observed in classical cyclin-Cdk complexes, with concomitant induction of kinase activity [19]. p35 and p39 each also has a conserved N-terminal glycine residue that becomes myristoylated, targeting the Cdk5 holoenzyme to membranes. In some contexts, p35 and p39 are cleaved proteolytically by calpain to produce the truncated derivatives p25 and p29, respectively. When these cleaved forms are bound to Cdk5, the kinase is hyperactivated due to reduced protein turnover. As the cleaved forms also lack the myristoylation site, Cdk5/p25 and Cdk5/p29 are cytoplasmic rather than membrane-associated, and therefore phosphorylate a different spectrum of cellular proteins [16]. p35 and p39 have different but overlapping distributions in the brain, suggesting that they are required for distinct functions *in vivo*, though they appear to be interchangeable in most experimental paradigms [20]. Cdk5 is also regulated by phosphorylation of the catalytic subunit, most notably at tyrosine 19 [21]. This phosphorylation further stimulates the kinase activity of Cdk5/p35. That is in contrast to traditional cyclin-Cdk complexes, whose kinase activity is reduced by phosphorylation at the homologous N-terminal tyrosine [21]. The stability of p35 and p39 themselves depend on the activation state of Cdk5. Upon activation by p35, Cdk5 phosphorylates p35 causing its degradation through the ubiquitin proteasome system (UPS), thus providing a negative feedback loop that acts as a critical control point for Cdk5 activity [22].

## Cdk5 and Synapse Function: Effects on the Synaptic Vesicle Cycle

**Figure 2:**
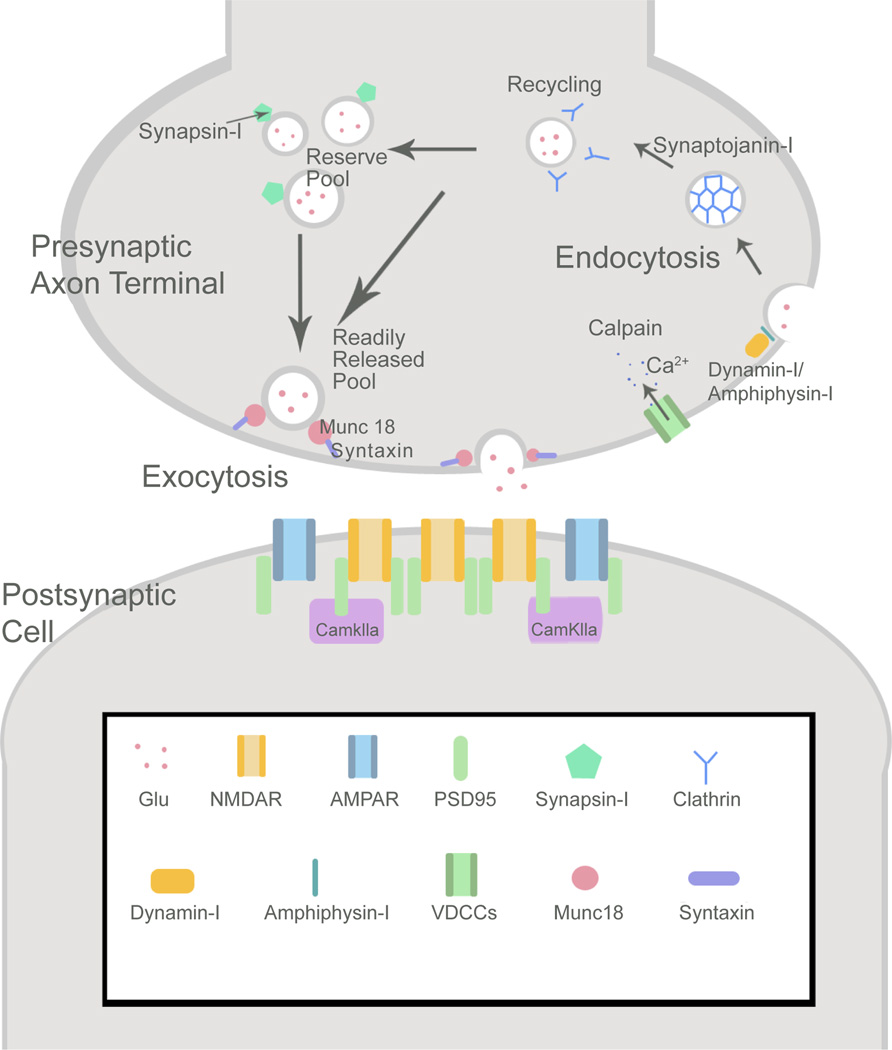
Targets of Cdk5 at pre- and post-synaptic sites Schematic representation of some binding partners and targets of Cdk5 at a glutamatergic synapse. Presynaptically, Cdk5/p35 interacts with SNARE proteins and their regulators such as Munc18 and syntaxin to facilitate neurotransmitter exocytosis. Synaptic proteins are recycled by dynamin-I and amphiphysin-I mediated endocytosis in clathrin-coated vesicles, and returned to the reserve or readily releasable synaptic vesicle pools. Cdk5 interacts with each of these proteins/receptor complexes. Postsynaptically, Cdk5 regulates NMDA/AMPA receptor clustering within the postsynaptic density through the scaffolding protein, PSD-95. Upon NMDA receptor activation p35 associates with CaMKIIα, potentially influencing signal transduction.

Cdk5 is a major regulator of synaptic function through its control of vesicle exocytosis and endocytosis (Figure 2). Indeed, increase in Cdk5 activity can silence the nerve terminal altogether [23]. Cdk5/p35 interacts with multiple targets to influence exocytosis via the SNARE (soluble NSF-attachment protein receptor) complex that is central to synaptic vesicle fusion and recycling. Cdk5 facilitates exocytosis through phosphorylation of Munc18, thus freeing syntaxin1A to form a SNARE complex and facilitate neurotransmitter release [24,25]. Cdk5/p25 phosphorylates another cyclin-dependent kinase, PCTAIRE1, enhancing its kinase activity [26] and thereby activating NSF (N-ethylmaleimide-sensitive fusion protein) [27]. Upon activation, NSF acts as a molecular chaperone that alters the conformation of, disables, and then recycles SNARE monomers for subsequent membrane fusion [27]. It is interesting that Cdk5 phosphorylation of PCTAIRE1 also impedes dendrite development, potentially linking a mechanism that regulates synaptic physiology with one that controls neuron structure directly [28].

Cdk5 uses multiple routes to modulate vesicle endocytosis, and although the targets are largely agreed upon, the outcomes of these interactions are often disputed. For example, Cdk5 phosphorylates synapsin-I, although whether this interaction increases or decreases synaptic transmission is unknown [29]. Further, Cdk5 constitutively phosphorylates amphiphysin I [30] and synaptojanin-I during resting states [31,32]. Cdk5 phosphorylates dynamin I, but there are conflicting reports on whether dynamin I phosphorylation by Cdk5 is essential for endocytosis [31] or interferes with it [32]. Beyond the direct effects these interactions have on vesicle uptake, it should be noted that they also provide an indirect input back upon vesicle exocytosis by controlling the vesicle pool.

Cdk5, and more specifically its balance with calcineurin, also directly determines the rate of vesicle recycling, with acute inhibition of Cdk5 serving to free resting vesicles into the recyclable vesicle pool. The dynamic between Cdk5 and calcineurin is further influenced by pre-synaptic activity, since chronic silencing of neuronal activity by tetrodotoxin (TTX) suppresses presynaptic Cdk5 activity [23].

## Cdk5 and Neurotransmitter Action

Cdk5 has profound effects on nervous system activity through altering the synthesis or receptor function of acetylcholinergic [33–35], catecholaminergic [36], and glutamatergic [9] systems of neurotransmission. Cdk5 is activated by acetylcholine (ACh) agonists and is required for ACh receptor clustering and signaling in motor axons and neuromuscular junctions, thus playing a necessary role in the formation and remodeling of neuromuscular synapses [33,37]. At the developing neuromuscular junction, transcription of the ACh receptor gene occurs at subsynaptic nuclei and is controlled by neuregulin-1 (NRG-1). Cdk5 activity is required downstream of the ErbB neuregulin receptor for neuregulin-induced up-regulation of ACh receptor gene expression [34]. Further, Cdk5 is required for Ach agonist-mediated AChR disassembly in post-synaptic regions that fail to make appropriate connections with presynaptic terminals [37]. Dispersal of such aberrant receptor clusters may occur through nestin/Cdk5 interaction [35].

Cdk5 regulates dopamine (DA) synthesis via the rate-limiting enzyme in catecholamine synthesis, Tyrosine Hydroxylase (TH). Through phosphorylation of serine 31 of TH, Cdk5 increases the stability and activity of the protein in the substantia nigra of the midbrain [36,38]. The physiological necessity for this interaction is demonstrated by diminution in the level of TH protein in Cdk5 knock-out mice [36]. Cdk5 further decreases DA signaling through phosphorylation of DARPP-32 (dopamine- and cAMP-regulated phosphoprotein of *Mr* 32 kDa), a signal transduction molecule that regulates the amount of dopamine signaling in neostriatal neurons [4,39]. The relationship between dopamine and Cdk5 is bi-directional, as dopamine receptors can also indirectly influence the localization and kinase activity of Cdk5. Thus, stimulation of the D1 dopamine receptor leads to increased intracellular Ca^2+^, inducing calpain proteolysis of p35 to p25, and leading to increased Cdk5 activation. This in turn induces hyperphosphorylation of tau producing signs of neurodegeneration, including cell death [40].

Regulation of glutamatergic transmission by Cdk5 is largely postsynaptic, and again involves a variety of molecular pathways. NMDA-type glutamate receptors are direct targets of Cdk5, with the kinase phosphorylating the NR2A subunit of NMDA receptors on Ser- 1232 to increase channel activity [5,12]. This was observed in CA1 of the rodent hippocampus, both in the context of induction of Long Term Potentiation (LTP) and also in the response to transient ischemia, as well as in cell culture (transfected human embryonic kidney 293 cells). In contrast, in the striatum, inhibition of Cdk5, rather than activation, enhances NMDA-mediated glutamatergic transmission [9]. In this context, however, the effect on NMDA currents arises from Cdk5- regulated dopaminergic signaling that indirectly modulates NMDA signaling, not directly from phosphorylation of the NMDA receptor itself. This further highlights the need to consider the complexities of the complete biological context when interpreting the sometimes paradoxical effects of Cdk5.

Beyond modification of the receptor itself, Cdk5 also regulates glutamatergic transmission at the level of structural proteins of the postsynaptic density. The post-synaptic scaffolding protein PSD-95 regulates clustering and density of ionotropic glutamate receptors, and is a substrate of Cdk5 [41,42]. PSD-95, in complex with PSD-93, contributes to the organization and maturation of the synapse [43,44]. Inhibition of Cdk5 increases the binding of Src to PSD-95, which decreases NMDA receptor endocytosis [45]. Ubiquitylation of PSD-95 by a ubiquitin E3 ligase is induced by Cdk5 and is another mechanism by which Cdk5 is involved in NMDA/AMPA receptor endocytosis at the post-synaptic density [46]. The up/down regulation of NMDA receptor trafficking accumulation on the plasma membrane is an important component of the dynamic synaptic changes that underpin plasticity.

## Cdk5 and Synaptic Homeostasis

Many of the synaptic effects of Cdk5 modulate neural plasticity and synaptic homeostasis. To protect against excessive excitation, pre-and post-synaptic neurons engage in a homeostatic process known as synaptic scaling, which is a form of plasticity that allows individual neurons to regulate their overall rate of firing action potentials [47]. During normal function, this scaling is thought to stabilize neuronal circuits and prevent run-away excitotoxicity. In an elegant series of experiments, Seeburg et al. [48] demonstrated that Cdk5 is required for scaling through its interaction with Polo-like kinase-2 (Plk-2). Plk-2 is an activity-regulated gene that contains a C-terminal Polo-box domain (PBD) known to modulate kinase activity, and they hypothesized it might play a role in synaptic homeostasis. Indeed, stimulation of hippocampal neurons transfected with a dominant interfering construct that prevents Plk-2 phosphorylation exhibited no evidence of scaling of synaptic potentials compared with un-transfected cells, indicating Plk-2 is required for downward scaling during chronic excitatory stimulation. Further, Plk-2 binds to spine-associated RapGAP (SPAR), a post-synaptic protein that interacts with PSD-95 and promotes dendritic spine formation, and this phosphorylation is necessary for SPAR degradation. Cdk5 further modulates this signaling complex through its action as a SPAR “priming” kinase. This precursor phosphorylation is necessary before SPAR can be degraded. By this mechanism, Cdk5 dampens synaptic strength during stimulation to avoid excessive excitation.

Another mechanism of PSD-linked, Cdk5-dependent synapse loss, in frontal cortical neurons, stems from the interaction of Cdk5 with the postsynaptic density scaffolding protein GKAP from the MAGUK family of proteins [49]. Soluble Aβ peptide caused increased phosphorylation of GKAP by Cdk5 that triggered ubiquitination and proteasomal degradation of GKAP, resulting in the disconnection of postsynaptic density proteins from the actin cytoskeleton. It is interesting that many studies of the stability and strength of the postsynaptic site converge on ubiquitin-dependent protein degradation as a common regulatory mechanism. Cdk5-dependent phosphorylation targets numerous proteins for degradation by the as well as regulating its activity [50–52].

## Behavioral Consequences of the Synaptic Functions of Cdk5

The net effect of Cdk5 phosphorylation of a multitude of synaptic targets is regulation of global organismal processes such as cognition and behavior. Hebbian Long-Term Potentiation (LTP) strengthens synapses and is presumed to be a central mechanism of learning and memory. Cdk5 activity clearly modulates LTP, but in just what way remains controversial. For example, inhibition of Cdk5 with roscovitine has been reported to block induction of LTP [5], while others claim there is no direct effect on LTP, and rather that roscovitine prevents the inhibition of LTP by amyloid-β peptide [53]. Moreover, mice with a null mutation of p35 exhibit a lower, not higher, threshold for LTP induction [54] but impaired LTD [55]. These different outcomes may in part reflect differences in the experimental protocols that were used, in terms of cell type, developmental stage, and even the time course of the experiment. For example, although transient expression of p25 enhances hippocampal LTP, prolonged expression led to impairment [56]. This likely arises from Cdk5 having unrelated effects on different processes with different time courses, such as acute effects on synaptic vesicle cycling and long-term effects on axon and dendrite structure (see below). As far as the molecular mechanism of Cdk5 effects on LTP are concerned, one proposed mechanism is through calcium-mediated association of p35 and p39 with the alpha subunit of CaMK II, a key mediator of LTP [57]. This association is stimulated in response to activation of NMDA receptors, though its functional significance remains uncertain.

Cdk5 function has profound effects on various forms of hippocampal learning and memory [6,7,58,59]. Much like the effects on LTP, however, despite general agreement on the necessity of Cdk5 for wild type memory, whether Cdk5 loss of function results in memory deficit or improvement is currently controversial. For example, a conditional Cdk5 loss-of-function mutation resulted in impairments in the formation and retrieval of hippocampus-dependent memories via cAMP signaling [59]. In contrast, however, Hawasli et al. [6] report that Cdk5 conditional knockout mice showed *enhanced* associative and spatial memory for hippocampus-dependent tasks, though no differences were observed in hippocampus-independent tasks. Indeed, Ris et al. [60] hypothesize that production of p25 in Alzheimer’s disease may act as a compensatory mechanism for early learning and memory deficits in the progression of the disease. It is conceivable that some of the complexity in interpreting effects of Cdk5 on memory formation arise from interference by prior memories. Work done by Sananbenesi and others [58] demonstrates that genetic and pharmacological inhibition of Cdk5 is necessary to extinguish preexisting associative memories, and, conversely, that increasing Cdk5 activity impairs extinction. Perhaps failure to extinguish old memories can interfere with formation of new ones. One way Cdk5/p25 may modify memory formation and extinction is through dysregulation of the histone deacetylase HDAC1 [61]. Inactivation of HDAC1 results in aberrant cell-cycle activation, DNA damage, and neurotoxicity [62], but is also important in the regulation of fear extinction [62]. Overall, although the relationship of Cdk5 to learning remains murky, Cdk5 undoubtedly affects hippocampal plasticity. Furthermore, the ability of this kinase to affect the formation and extinction of fear memories makes Cdk5 an intriguing target for cognitive and emotional disorders.

## Structural Roles of Cdk5

**Figure 3:**
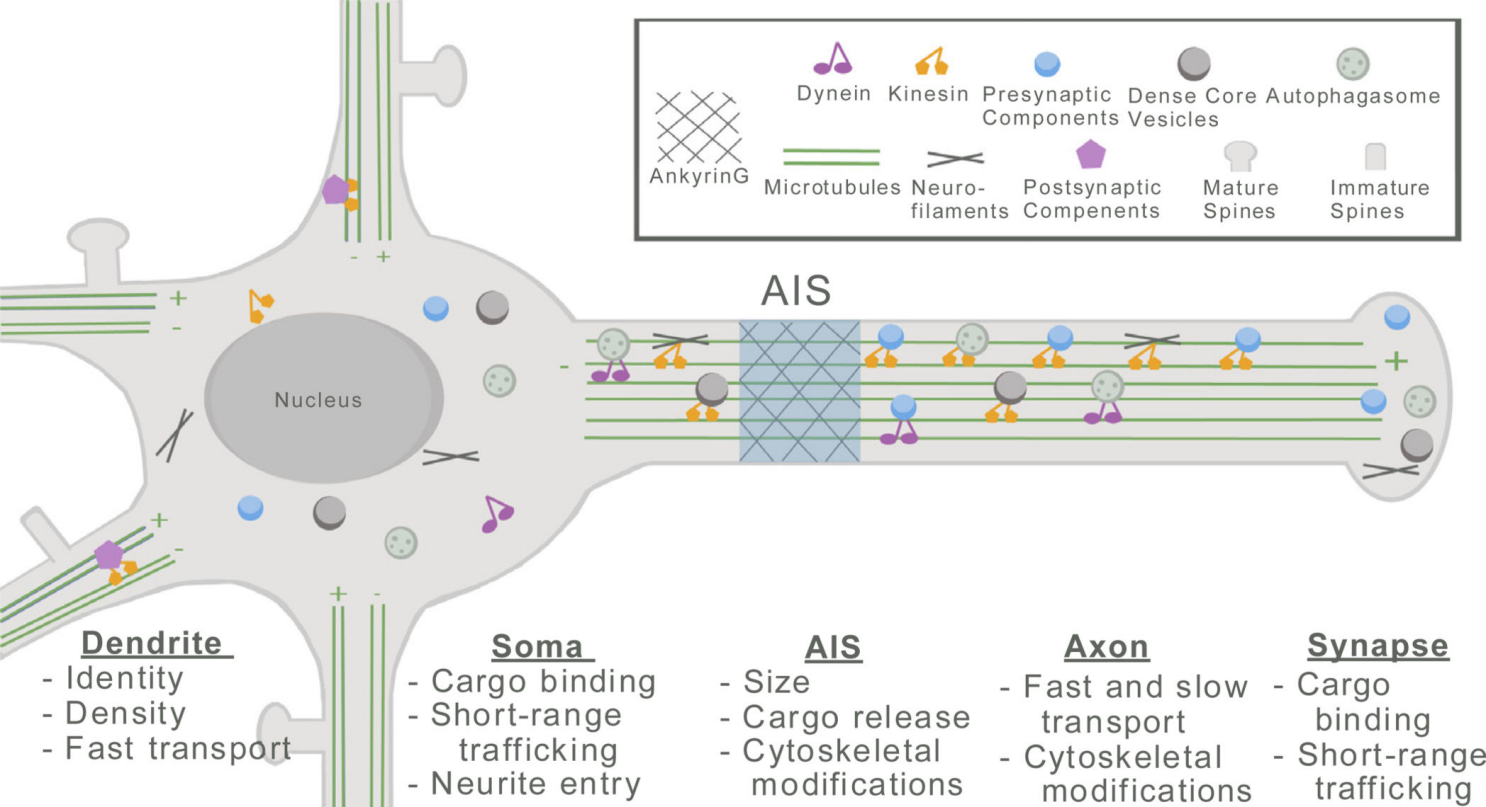
Roles of Cdk5 in neuronal structure and trafficking Schematic of a typical neuron, with spatially localized Cdk5-regulated functions listed under the cellular compartment in which they occur. Symbols referring to specific molecular and cellular components are defined in the boxed key at the top. Microtubule polarity is indicated by (+) and (−) symbols. Note that positioning of the components in neuronal processes typically reflects a balance of anterograde and retrograde trafficking. Most components are therefore shown in association with both dynein and kinesin motors.

Beyond regulating the composition and transmission properties of individual synapses, Cdk5 also modulates the overall structure and sub cellular organization of neurons. This includes the polarization of the neuron, compartmentalization of the axon and trafficking of its components, the structure of dendrites and synapse density (Figure 3). A growing body of evidence demonstrates the action of Cdk5 in neuronal maturation and structural plasticity, with implications for disorders of neural activity and neurodegenerative disease.

## Cdk5 and Initial Neuronal Polarization

An early step in organizing a mammalian neuron is the selection of one of its neurites to be the nascent axon while the others differentiate into dendrites. Axon formation is based on tightly regulated events of cytoskeletal reorganization [63] in response to extracellular cues and intracellular signaling [64,65]. Axon selection in hippocampal neurons and developing cortical neurons is regulated by Cdk5 through modulation of the interaction between Axin and GSK-3β, two major regulators of axon specification [66]. Axin (Axis inhibitor) is a scaffold protein of the Wnt signaling pathway that mediates axon initiation by stabilizing microtubules (MTs) through the control of GSK-3β localization. Cdk5-mediated phosphorylation of Axin inhibits its interaction with GSK-3β, stabilizing the microtubule network and promoting axon formation.

The possibility of a very different and indeed opposite, effect on axon formation for Cdk5 is suggested by its interaction with the repulsive guidance molecule Semaphorin3A (Sema3A) [67]. Sema3A signaling decreases protein kinase A (PKA) activity and down regulates PKA-dependent phosphorylation of the axonal determinants LKB1 and GSK-3β, thereby suppressing axon formation and enhancing dendrite specification in cultured hippocampal neurons and cortical neurons *in vivo*. Separate studies, however, showed that Sema3A function is mediated by Cdk5 [68]. Cdk5 phosphorylates Collapsin Response Mediating Protein-2 (CRMP2) that is a part of the Sema3A intracellular signaling pathway. CRMP2 phosphorylation, first by Cdk5 and then by GSK3β reduces its affinity to tubulin in dorsal root ganglion neurons and promotes Sema3A-induced growth cone collapse in cerebral cortex [68,69]. This raises the possibility that Cdk5 can either promote axon formation or inhibit it, depending on the balance of extracellular signaling. Additional experiments will be necessary to test this conjecture.

## Intra-axonal Compartmentalization

Axons are not just featureless shafts. They have a complex internal organization that is fundamental to neuronal function and maintenance. The Axon Initial Segment (AIS) is the site of action potential initiation, and it acts as a gatekeeper in intra-axonal traffic, segregating the somatodendritic vs axonal compartments [70]. Moreover, the AIS is a central player in the plasticity of neuronal function. Thus, chronically increased neuronal activity caused the AIS to shift in a distal direction in cultured hippocampal neurons [71]. Recent experiments in central brain neurons of *Drosophila* show that Cdk5 is a dose-dependent regulator of AIS size [1]. Reduction or elimination of Cdk5 activity caused severe reduction in AIS length, and hyperactivation of Cdk5 (by over-expression of p35) led to extension of the AIS. In this system, the length of the AIS was controlled via positioning of its distal boundary while the position of the proximal boundary was unchanged.

The unique electrophysiological properties of the AIS, particularly initiation of action potentials, rely on selective enrichment of specific subtypes of sodium, potassium and calcium channels, and exclusion of others [70]. In mature hippocampal neurons, Cdk5 regulates the phosphorylation dependent targeting of voltage-gated Kv1 potassium channels to the AIS [72–74]. Cdk5-mediated phosphorylation of the auxiliary subunit Kvβ2 disrupts the interaction of Kv1 complexes with the microtubule plus end-tracking protein EB1, thus allowing Kv1 localization to the plasma membrane. Conversely, acute inhibition of Cdk5 increased the intra-axonal concentration of EB1 protein, and of Kv1-Kvβ2 complexes in association with MTs at the AIS, locally limiting channel insertion into the plasma membrane. Additionally, Cdk5 regulates the balance between associations of EB1 with the AIS *vs* other regions of the cell. For another potassium channel Kv2.1, Cdk5-mediated phosphorylation regulates the steady state level of channel clustering in somatic and axonal compartments [75]. This phosphorylation was activity-induced and reversible, and showed a potential regulatory role of Cdk5 in determining the intrinsic excitability of the neuron [74,75].

Propagation of action potentials in myelinated neurons depends on the function of ion channels clustered at nodes of Ranvier [76,77]. Clusters of Kvβ2 at the node of Ranvier colocalize with Cdk5 in the mouse sciatic nerve [74]. Moreover, Cdk5 immunostaining is present at the node and the paranode, and is enriched at the juxtaparanode. Given the many molecular similarities between nodes and the AIS, it seems worthwhile to ask whether the localization of Cdk5 at the nodes has a function in supporting or modulating action potential propagation, though this has not been tested.

## Dendritic Development: Arborization and Spine Morphogenesis and Maintenance

Dendrites are dynamic structures. Their morphology and organization control how neurons process information [78], and structural changes in dendritic arborization and dendritic spine morphology underlie learning and memory formation [79,80]. In mammalian neurons, Cdk5 regulates dendrite development by promoting signaling from many extracellular cues as well as by modulating cytoskeletal dynamics [81]. Some of the effects of external cues may be mediated by S-nitrosylation of Cdk5 itself, with consequent reduction of Cdk5 kinase activity [82]. Lack of Cdk5 function causes defects in dendrite development during embryogenesis and in the adult. In cortical neurons of Cdk5-deficient mice, these were correlated with reduced expression of the microtubule-associated protein MAP2 [83]. In addition to modulating the pattern of dendritic arborization, Cdk5 also has a specific role at a later step in dendrite morphogenesis, maturation of dendritic spines. Cdk5 inhibition impaired the development of immature, spiky dendritic spines into large “mushroom-shaped” spines and reduced the number of such mature “mushroom” spines even in correctly targeted dendrites in rodent hippocampal granule cells [84]. Since establishment of stable excitatory synapses is physically supported by these mushroom-shaped spines [85,86], this identifies another mechanism by which Cdk5 promotes synaptic transmission and LTP.

Cdk5 controls dendritic spine density using a variety of molecular mechanisms. Cdk5 phosphorylation of the WAVE1 protein inhibits its interaction with the Arp2/3 actin polymerization complex, and consequently inhibits spine morphogenesis [84–87]. This process is activity-regulated, with NMDA-dependent depolarization of the cell leading to degradation of p35, thus releasing the inhibition [88]. Cdk5 also stimulates spine retraction in hippocampal neurons by regulating signaling downstream of Eph receptor A4 (EphA4) [89]. Upon stimulation by ephrin A1, EphA4 recruits Cdk5 and phosphorylates it on tyr15, thereby enhancing Cdk5 activity. Cdk5, in turn, phosphorylates the Eph effector Ephexin1, a guanine nucleotide exchange factor that regulates actin cytoskeletal dynamics by stimulating RhoGTPase [89]. Inhibiting Cdk5 activity thus prevents ephexin1 phosphorylation and blocks ephrin-A1–induced spine retraction. Cdk5 also phosphorylates another Rho GEF, kalirin-7, increasing its activity to stabilize the spine. Mutation of the necessary Cdk5 phosphorylation site on kalirin-7 results in aberrant spine morphology [90].

## Traffic Control

Neuronal excitability and neurotransmission are profoundly sensitive to long-range intracellular trafficking. Cdk5 regulates this process by modifying motors, their cargo, the scaffolding proteins that connect them, and the cytoskeletal proteins themselves, as we now discuss.

## Cdk5 and Motor Proteins

Outward transport from the soma to the distal tips of the axon and dendrites is largely powered by the diverse family of kinesins (KIFs) [91,92]. Kinesins have a conserved motor domain and typically move toward the “plus” ends of microtubules. Cdk5 was shown to stimulate kinesin-based anterograde motility in axoplasm of squid giant axons by regulating the activity of GSK3 [93]. Cdk5 inhibits protein phosphatase 1 (PP1), thereby activating GSK3. This, in turn, phosphorylates kinesin light chains, causing dissociation of transported cargo from kinesin in the axonal compartment. The authors suggested that the regulated release of cargo allows neurons to target organelle delivery to specific subcellular compartments [93]. In this system Cdk5 affected only anterograde but not retrograde transport [93,94].

Cdk5 has a well-established role in regulating retrograde trafficking in the axon by controlling the activity of dynein as well as its interaction with different cargos. Cdk5 phosphorylates NUDEL (Ndel1), a protein that binds both dynein and the dynein interacting protein, LIS1 [95,96]. Phosphorylation by Cdk5 both releases an inhibitory effect of Ndel1 on the LIS1/dynein complex, and also stimulates the ability of LIS1 to enhance the transport capacity of dynein, perhaps by increasing the force produced by the motor to facilitate cargo transport through the viscous cytoplasm. Consequently, movement of acidic organelles in axons of adult rat sensory neurons is impaired upon inhibition of Cdk5 activity [97,98]. Cdk5, Ndel1 and LIS1 also interact to promote dynein-dependent radial migration of cortical neurons, and inhibition of this interaction underlies the developmental brain abnormality, type I lissencephaly, as well as modifying intracellular NUDEL distribution and causing swellings along neuritic processes in embryonic cortical cultures [95,96].

Studies of Cdk5 function in *C. elegans* have offered particularly rich opportunities for dissecting some of the many ways that this kinase regulates axonal trafficking. For example, in cholinergic DA9 motoneurons Cdk5 cooperates with the PCTAIRE kinase (PCT-1) to suppress retrograde transport by negatively regulating dynein function [99]. In this way, the dominance of kinesin-3-dependent anterograde transport is reinforced in these neurons. Thus, presynaptic vesicles were mislocalized to the dendritic compartment in *Cdk5* mutants and the number of retrogradely-moving vesicles was significantly increased in *PCT-1, Cdk-5* double mutants. That this reflects a shift in a dynamic balance of anterograde *vs* retrograde motility is demonstrated by the observation that mutations in the genes encoding dynein heavy chain or Ndel can suppress the mislocalization phenotype of *Cdk5* mutants.

In other *C. elegans* neurons, Cdk5 interacts with PCT-1 in different ways to control the balance of anterograde and retrograde transport. In a subset of cholinergic neurons PCT-1 was shown to be a downstream target of Cdk5 rather than acting redundantly [27]. In yet a third setting in the worm, GABAergic DD motoneurons, the CyclinY (CYY-1)/PCT-1 and p35/Cdk5 kinases act sequentially to control distinct steps in trafficking of synaptic components [100]. These neurons remodel their synapses during development by eliminating early larval synapses that are proximal to the cell body and reusing the synaptic components to construct definitive synapses more distally. CYY-1/PCT-1 is required for the disassembly of the original synapses, while p35/Cdk5 first stimulates kinesin-3 to promote delivery of synaptic material to distal portions of the axon, and then modifies dynein activity to redistribute these materials along the axon in a proximal direction at a later step in synapse formation.

Actin-based mechanisms of polarized transport also exist in neurons, particularly for short-range trafficking, and are also modulated by Cdk5 [92,101]. These have recently been reviewed in detail by Lalioti and co-authors in the context of synaptic vesicle cycling and cell migration [102]. We note, however, that the effects of Cdk5 are mediated through modulation both of myosin function and of the dynamics of the actin cytoskeleton itself, executed through the Rho GTPase Cdc42 and its kinase partner, Pak-1 [101].

## Scaffolding Molecules

The specificity of neuronal trafficking relies heavily on adaptor and scaffolding proteins that link cargos to their appropriate motors, and Cdk5 regulation is often directed at these interactions. In *C. elegans*, Cdk5 promotes trafficking of glutamate receptors (GLR-1), probably acting at an early stage in the secretory process, in the soma [103]. It is thought that this is mediated, in part, via Cdk5-dependent phosphorylation of the PDZ-containing scaffolding protein LIN-10/Mint-1. LIN-10/Mint-1 can act as a cargo adaptor protein supporting association of neurotransmitter receptors with kinesin motors and promote trafficking from ER and Golgi to the synapse [103]. By this model Cdk5 promotes GLR-1 anterograde traffic by controlling the abundance of LIN-10/Mint-1, and thus its ability to promote GLR-1 entry into neuronal processes.

Cargo selective effects of Cdk5 on transport are also apparent in the specific trafficking of neuropeptide filled Dense Core Vesicles (DCVs) in cholinergic DB motoneurons of *C. elegans* [104]. Cdk5 activity modified DCV distribution in both axon and dendrite, primarily by modulating kinesin-3 dependent anterograde transport. Synaptic vesicle distribution was not affected. The molecular mechanisms are not completely clear, but probably are not due to direct regulation of the kinesin-3 or dynein motors. Goodwin and co-authors [104] suggested that Cdk5-dependent phosphorylation of a cargo adaptor protein in the soma might work as a switch controlling association with kinesin3 *vs* dynein and thus promoting polarized trafficking in axons and preventing dynein dependent trafficking into dendrites.

In several cases, Cdk5-regulated adaptor proteins are implicated in neurological disorders making them of particular interest. Huntingtin (Htt), the protein mutated in Huntington’s disease, acts as a scaffolding protein that facilitates interaction between dynein and kinesin motors and their cargo, and phosphorylation of Htt by Akt kinase regulates the directionality of Htt-dependent transport [105,106]. Cdk5 also phosphorylates wild type Htt protecting it from caspase cleavage, and inhibition of Cdk5 enhances accumulation of mutated polyQ-expanded Htt in protein aggregates [107]. Cdk5 can also inhibit Htt aggregate formation by disrupting microtubules that are required for the formation of Htt inclusions [108].

Another Cdk5-associated adaptor protein is Disrupted in schizophrenia 1 (DISC1), which has multiple functions including microtubule-mediated transport and regulation of the structure and composition of dendritic spines [109]. Mutations in DISC1 are strongly associated with psychiatric disorders, such as schizophrenia, major depression, bipolar disorder, and autism [109]. Two-hybrid screens and biochemical experiments revealed that DISC1 binds Cdk5 [110], and the DISC1 interactome includes two other proteins that are associated with Cdk5 and that interact functionally with DISC1 in the migration of embryonic cortical neurons, NDEL and DIXDC1 (Dix domain containing 1) [111]. The observation of both physical and functional interactions among this group of four proteins that share common biological functions makes them an attractive target for investigating the mechanisms that underlie DISC1-associated psychiatric disorders.

## Functional Modifications of the Microtubule Cytoskeleton by Cdk5

Post translation modifications of MTs control their stability and availability for interactions with other molecules, including motors. Cdk5/p35 binds tubulin, promoting microtubule polymerization and stability [98,102,112]. This stabilization of MTs can be abolished by competition with calmodulin (CaM) in a calcium dependent manner suggesting a switchable balance between the Cdk5 and CaMKII signaling pathways [113]. Cdk5 also regulates other signaling pathways that have an effect on MT stability. Tubulin acetylation leads to microtubule stabilization and resistance to depolymerizating agents, as well as promoting binding of molecular motors. SIRT2, a member of the Sirtuin family of NAD(+) dependent deacetylases, targets alpha-tubulin and inhibits neurite growth in hippocampal neurons [114]. Cdk5-mediated phosphorylation of SIRT2 at Ser-331 inhibits the catalytic activity of the protein and thus increases alpha-tubulin acetylation. Other protein deacetylases, such as HDAC6, can also act downstream of Cdk5 and show patterned distribution in axons, potentially offering another pathway for Cdk5 to regulate the modification state of microtubules.

Cdk5 also regulates the microtubule cytoskeleton through its effects on Microtubule Associated Proteins (MAPs) and intermediate filaments (IFs). Two of the most prominent MAPs, MAP2 and tau, are well-established targets of CDK5 activity [98,115,116]. MAP2 is located in the somatodendritic compartment of mature neurons, while tau is largely axonal [117]. MAP2 phosphorylation by Cdk5 stabilizes MTs, though the physiological significance of this modification remains unclear. In contrast, phosphorylation of tau by Cdk5 reduces its affinity for microtubules, thereby reducing microtubule stability [118,119]. Moreover, tau can control neuronal traffic by interacting with kinesins independently of its effects on microtubules [120,121]. The effect of Cdk5 phosphorylation of tau has been studied intensively due to the accumulation of hyperphosphorylated tau in the intracellular tangles that are a hallmark of Alzheimer’s disease [122–124]. It remains controversial whether the pathological effects of phospho-tau are due to its aggregation *per se*, or to the effects of the modification on wild type functions of tau in processes such as trafficking. In this context, it is important to remember that neurotoxic effects of altered Cdk5 activity have been associated with changes in trafficking and clustering of various cellular organelles, including ER and mitochondria [125,126].

Neurofilaments (NFs) also have multiple potential Cdk5 phosphorylation sites on their C-terminal tails [127]. The extensive modification of NF by Cdk5 is associated with redistribution of the protein from its wild type axonal localization to accumulation in the cell body in the neurodegenerative state [128]. The C-terminal phosphorylation of the neurofilament heavy chain (NF-H) can redirect NF from binding kinesin to binding dynein, perhaps contributing to this redistribution of the protein [127]. Moreover, it has been proposed that C-terminal phosphorylation of NF plays a role in forming cross-bridges between neurofilaments and microtubules, immobilizing neurofilaments, stabilizing the axon and slowing axonal transport, potentially affecting the transport of other microtubule-dependent cargo as well as NF itself [127].

Thus, Cdk5 regulates neuronal trafficking at a variety of levels, and in ways that are highly specialized depending on cell type, cellular compartment and physiological state. Many of these activities of Cdk5 are undoubtedly connected to its roles in neuropathology, as we will discuss below.

## Cdk5 and Pathology

In the final section of this review we will consider how disruptions in the function of Cdk5, and particularly those functions related to neuronal excitability, neurotransmission, protein trafficking and the compartmentalization of the neuron, contribute to neurological disease.

## Synaptic Functions of Cdk5: Implications for Neuropathology and Disease

Given the many ways in which Cdk5 modulates neural structure and activity, it is not surprising that alterations of Cdk5 activity are associated with an equally broad range of neurological disorders. The neurotransmission effects of Cdk5 have many impacts on neural function, sometimes in complex and unexpected ways. For example, Cdk5 can alter dopaminergic transduction, and, in turn, DA and NMDA receptor activity can cause over activation of Cdk5 that leads to toxicity and cell death. Paoletti et al. [11] propose a model of HD where mutant huntingtin protein increases activation of DA and NMDA receptors, leading to dysregulated intracellular Ca2+ and overactivation of calpains [129]. Calpains then increase the conversion of p35 to p25 [130], which in combination with NMDA-potentiated DA1-phosphorylation of Cdk5, phosphorylates tau and initiates striatal cell death. This proposal is further supported by observed elevations in Cdk5 levels in HD knock-in mice and HD human brains [11]. Sometimes the relationship is more complex, however. While Cdk5 typically limits excitatory glutamatergic transmission, for example, once an excitotoxic event is underway Cdk5 activity can intensify the consequent degeneration of hippocampal neurons [13]. It was hypothesized that the Cdk5-mediated activation of NMDA receptors is an underlying cause of ischemia-induced damage in CA1 pyramidal neurons [12] and expression of a Cdk5 dominant negative mutant protected neurons from such damage. Further, Cdk5 hyperphosphorylates tau in the wake of ischemic events that induce formation of p25, potentially initiating cellular degeneration [131], and thus exacerbating neuronal damage and loss. These findings indicate a dichotomous role for Cdk5 in either preventing or exacerbating excitotoxic events such as epilepsy and ischemia.

Emotional disorders may also be linked to Cdk5, both through its generalized effects on neuronal excitability and more specifically those on DA metabolism and function. Dopamine and the dopaminergic nuclei (substantia nigra pars compacta, nucleus accumbens, and ventral tegmental area) are important in reward signaling. Reward or reinforcement cause a behavior to increase in intensity or frequency and is critical to the development of habit. Almost all addictive drugs cause increased dopamine release and this signaling is an important neural basis of substance abuse. Cdk5 is implicated in regulating the neural processes underlying cocaine addiction through its effects on dopaminergic neurotransmission [132]. Chronic cocaine administration increases striatal Cdk5 production and activity, which interacts with dopaminergic targets mentioned previously, including DARPP-32 [133] and tyrosine hydroxylase [38]. Infusion of Cdk5-inhibitors in the nucleus accumbens prior to cocaine administration resulted in increased locomotor function. Cdk5 activity within the basolateral amygdala is critical for memory consolidation and reconsolidation of reward-associated environmental cues [134]. These findings suggest a role for Cdk5 as a negative regulator of the behavioral and biochemical effects of cocaine exposure.

In addition to drug-based models of Cdk5-mediated emotional disorders, p35 loss-of-function has been proposed as a model of hyperactivity [135], and through its effects on DA signaling, dysregulation of Cdk5 has been linked as a contributor to ADHD pathology [14]. Administration of a drug used to treat ADHD in humans, atomoxetine, decreased impulsivity in adolescent rats and was associated with decreased Cdk5 mRNA expression [136]. These findings, in conjunction with those describing the role of Cdk5 in hyperactivity and ADHD, argue for a role of Cdk5 in impulsivity, reward, and addiction.

## Axon Trafficking and Neuropathology

Disruption of ion channel trafficking is often associated with neurological disorders including epilepsy, ataxia, pain and autism [137]. Consistent with this, Cdk5 contributes to neural disease in a host of ways related to its control of the polarized structure of the neuron and the trafficking of neuronal components. For example, the evidence linking Cdk5 to the functional organization of the AIS takes on added meaning in the light of recent data implicating the AIS in the pathology of Angelman syndrome and epilepsy [138,139].

Among axonopathies, amyotrophic lateral sclerosis (ALS) has probably the clearest association with axon transport as the motor neurons affected by the disease have unique characteristics such as very long axons and high energy metabolism [140]. ALS pathology is characterized by progressive degeneration of axons of motor neurons with accumulation of inclusion bodies containing phosphorylated NFs, SOD-1, TDP-43 and other proteins [140], leading to muscle atrophy and paralysis [141].

Alteration of Cdk5 function was shown in motor neurons in an SOD1-ALS mouse model [142]. ALS-type mutations in the *SOD-1* gene caused Cdk5 mislocalization in the cell and elevated activity of Cdk5 due to a changed p35/p25 ratio, and it caused hyperphosphorylation of tau and NFs. Previous studies had shown reduction in both fast and slow anterograde axonal transport of NFs and other cytoskeleton proteins in SOD1-ALS neurons. This was accompanied by accumulation of NFs and NF inclusions in the perikarya and proximal axon of motor neurons and reduced levels of NFs in the distal axon [143,144]. Given the highly regulated topographic regulation of NF phosphorylation in the soma and the axon, together with the differential effects of Cdk5 on transport in different neuron compartments in wild type neurons, this raises the possibility of Cdk5 involvement in early events of the traffic impairment in ALS pathology. Remarkably, perikaryal accumulation of NF in this model seemed to be associated with reduced, rather than enhanced accumulation of phospho-tau, leading the authors to suggest that formation of inclusions may be a protective mechanism employed by the cell [142].

Morphologically, transport defects often are manifested as accumulation of materials in the soma and neurites accompanied by swollen axon segments or spheroids [143–146]. Analysis of varicosities or swellings of the axon observed in many animal models of neurodegenerative diseases have shown that they contain abnormal organelles such as swollen mitochondria in ALS and Parkinson, and synaptic vesicles and protein aggregates in Alzheimer’s Disease. Cells eliminate these abnormal organelles and large protein aggregates by autophagy [147].

Recent results implicate Cdk5 activity in regulation of autophagy, though without addressing whether subcellular localization plays a role in Cdk5-dependent autophagy activation or inhibition. Loss of Cdk5 activity causes age-dependent accumulation of autophagosomes in the *Drosophila* central brain at a level exceeding that in physiologically matched controls [148]. In a mouse cell culture model of AD, Cdk5/p25 phosphorylated a critical component of the autophagy core complex, VPS34 [149]. VPS34 is a class III phosphatidylinositol-3 kinase that is involved in multiple vesicular trafficking events [150]. Cdk5-mediated phosophorylation of VPS34 interrupted its interaction with the autophagy protein Beclin1 and blocked induction of autophagy in response to starvation stress. These authors suggested that abnormal activation of Cdk5 in AD could negatively regulate autophagy and lead to cell death [149]. In contrast, the opposite effect on autophagy was observed in two models of Parkinson pathology, one using injection of MPTP into mice, and another employing an *alpha-synuclein* mutation [151]. In these cases, Cdk5 activity was required to activate autophagy by phosphorylation of endophilinB1 that in turn recruited another key component of the autophagy core complex, the UVRAG protein. This mechanism of autophagy induction increased cell death specifically under starvation stress. It is interesting that UVRAG is preferentially localized to late endosomes while the VPS-34-Beclin1 complex has preference for isolation membrane/phagophore during starvation. Perhaps the difference in subcellular localization is relevant to the opposite directionality of the effect of Cdk5 in these two cases, though we note that different cell types were also employed in the two experiments.

The question of whether autophagy enhances or suppresses neurodegeneration has been contentious. Autophagy can clearly be part of the mechanism of pathological degeneration, but just as clearly, lack of autophagy can trigger degeneration by the failure to remove dysfunctional mitochondria and toxic aggregates. It seems likely that the contradictory effects of Cdk5 on degeneration *via* its regulation of the autophagy pathway stem in part from this dual nature of autophagy itself, and will depend on the details of a specific pathological context. It may also be relevant that autophagy has quite different properties in the axon *vs* the soma. The abundance of autophagy core complexes at the synapse and the trafficking dynamics of the process depend strongly on their interaction with both kinesin and dynein motors in primary neuronal culture [152]. It is plausible that depending on the pathological condition Cdk5 may have a stronger effect on one side of the autophagic pathway or the other, depending on the predominance of its various interactions with molecular motors. It will therefore be interesting to determine whether compartment specific regulation of neuronal traffic and autophagy regulation by Cdk5 are related.

## Conclusion

Neuronal physiology depends on a series of balancing acts: excitation *vs* inhibition, stability *vs* turnover, transmitter release *vs* recycling, transport towards *vs* away from the soma and many others. Many neural disorders are linked to the failure of one or more of these delicate equilibria, and in a remarkable number of cases, Cdk5 plays a central role in the establishment and maintenance of the necessary physiological balance. Here we have reviewed a few illustrative cases, particularly those associated with neurotransmission and the affiliated processes of neuronal subcellular organization. Exploiting Cdk5 in the treatment of neural disorders and disease will require a nuanced understanding of the subtle physiology to which it contributes.
